# Conformational Switching in Bcl-xL: Enabling Non-Canonic Inhibition of Apoptosis Involves Multiple Intermediates and Lipid Interactions

**DOI:** 10.3390/cells9030539

**Published:** 2020-02-26

**Authors:** Victor Vasquez-Montes, Alexander Kyrychenko, Mauricio Vargas-Uribe, Mykola V. Rodnin, Alexey S. Ladokhin

**Affiliations:** 1 Department of Biochemistry and Molecular Biology, The University of Kansas Medical Center, Kansas City, KS 66160, USA; vvasquez@kumc.edu (V.V.-M.); a.v.kyrychenko@karazin.ua (A.K.); mauricio.vargasu@gmail.com (M.V.-U.); mrodnin@kumc.edu (M.V.R.); 2 Institute of Chemistry and School of Chemistry, V. N. Karazin Kharkiv National University, 4 Svobody Square, 61022 Kharkiv, Ukraine

**Keywords:** Bcl-2 proteins, BH4 domain, apoptotic regulation, conformational switching, protein-membrane interactions, Fluorescence Spectroscopy, Fluorescence Correlation Spectroscopy (FCS), Single Molecule FRET

## Abstract

The inhibition of mitochondrial permeabilization by the anti-apoptotic protein Bcl-xL is crucial for cell survival and homeostasis. Its inhibitory role requires the partitioning of Bcl-xL to the mitochondrial outer membrane from an inactive state in the cytosol, leading to its extensive refolding. The molecular mechanisms behind these events and the resulting conformations in the bilayer are unclear, and different models have been proposed to explain them. In the most recently proposed non-canonical model, the active form of Bcl-xL employs its N-terminal BH4 helix to bind and block its pro-apoptotic target. Here, we used a combination of various spectroscopic techniques to study the release of the BH4 helix (α1) during the membrane insertion of Bcl-xL. This refolding was characterized by a gradual increase in helicity due to the lipid-dependent partitioning-coupled folding and formation of new helix αX (presumably in the originally disordered loop between helices α1 and α2). Notably, a comparison of various fluorescence and circular dichroism measurements suggested the presence of multiple Bcl-xL conformations in the bilayer. This conclusion was explicitly confirmed by single-molecule measurements of Förster Resonance Energy Transfer from Alexa-Fluor-488-labeled Bcl-xL D189C to a mCherry fluorescent protein attached at the N-terminus. These measurements clearly indicated that the refolding of Bcl-xL in the bilayer is not a two-state transition and involves multiple membranous intermediates of variable compactness.

## 1. Introduction

**Figure 1:**
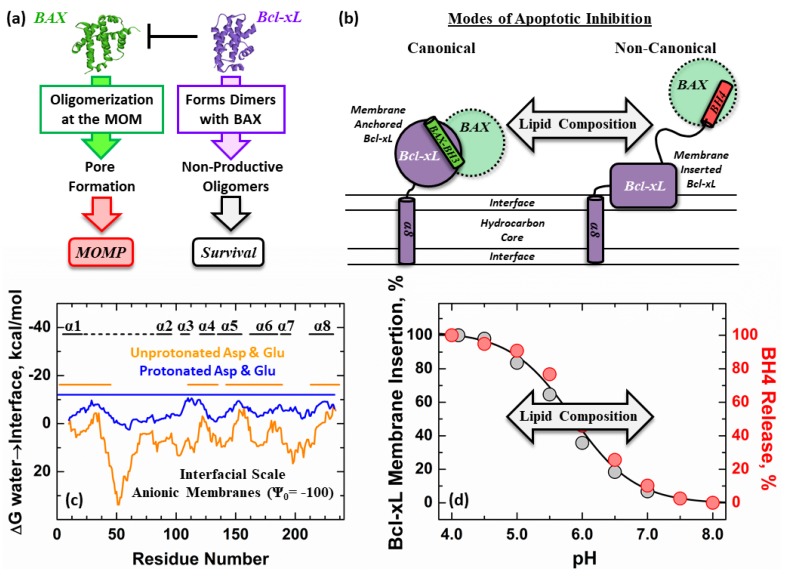
Conformational switching of Bcl-xL in membranes, resulting in conversion from canonical to non-canonical forms of apoptotic inhibition. (**a**) The anti-apoptotic protein Bcl-xL (purple) binds to the pore former BAX (green) to block the permeabilization of the mitochondrial outer membrane (MOM) and prevent apoptosis [3,4]. (**b**) Several molecular mechanisms, involving both membrane-anchored and membrane-inserted Bcl-xL, have been proposed to explain this process. The canonical mode (left) relies on the interaction of anchored Bcl-xL with the BH3 helix of BAX [7]; while in the non-canonical mode (right), BAX binds the N-terminal BH4 helix of refolded and inserted Bcl-xL. Lipid composition is hypothesized to modulate the transition between both inhibitory modes by facilitating the conformational switch between different conformations of Bcl-xL in the bilayer. (**c**) Bcl-xL hydropathy plot is presented for the two cases of either unprotonated (orange) or protonated (blue) titratable sidechains (D and E). This analysis was made using a modified version of the MPEx (http://blanco.biomol.uci.edu/mpex/) web tool [11], which accounts for both hydrophobic and electrostatic interfacial interactions [12]. The calculations were made assuming an approximate membrane surface potential (Ψ_0_) of -100 mV for a 1TOCL:2POPC bilayer, as described in Vasquez-Montes et al., 2019 [13]. Color-coded horizontal lines above the plot represent the regions of Bcl-xL predicted to interact with anionic membranes. The analysis showed a significant increase in the regions predicted to partition to the interface of anionic bilayers under protonating conditions with the largest effect observed for the unstructured α1-2 loop connecting the N-terminal BH4 (α1) helix to the rest of Bcl-xL. (**d**) Illustration of the lipid modulation of protonation-dependent membrane insertion and refolding of Bcl-xL from previously published data [13]. Relative insertion of Bcl-xL (grey symbols) is accessed by changes in fluorescence intensity of NBD (7-Nitrobenz-2-Oxa-1,3-Diazol-4-yl) attached to the N175C mutant. The refolding of Bcl-xL (red symbols) is accessed by steady-state FRET measurements of the release of its N-terminal BH4 helix. TOCL: 1,1,2,2-tetraoleoyl-cardiolipin; POPC: palmitoyl-oleoyl-phosphatidylcholine.

The anti-apoptotic protein Bcl-xL is a member of the Bcl-2 family of apoptotic regulators [1,2]. Its role in the cell is to block the mitochondrial outer membrane permeabilization (MOMP) caused by pro-apoptotic Bcl-2 proteins (i.e., BAX) (Figure 1a, green) [3,4]. The prevailing Embedded Together model of MOMP regulation postulates that membrane interactions are critical for both pro- and anti-apoptotic activities of Bcl-2 proteins [5,6]. Bcl-xL is expressed in the cytosol in an inactive state and must redistribute to the MOM where it refolds to become active. Bcl-xL promotes cell survival by inhibiting the formation of multimeric BAX pores and forming non-productive Bcl-xL/BAX heterodimers at the MOM (Figure 1a, purple) [7,8]. Recent evidence suggests that Bcl-xL is also a target of BH3-only apoptotic triggers [9,10].

The high-resolution structure of soluble Bcl-xL (Figure 1a) has been solved by both X-ray crystallography and NMR [14], revealing a helical fold typical for many Bcl-2 proteins [15]. In addition to this soluble conformation, at least two distinct conformations have been identified in membrane environments: “anchored” (Figure 1b, left) and “inserted” (Figure 1b, right). The structure of the anchored conformation reconstituted into nanodiscs has been solved by NMR, and it consists of a transmembrane C-terminal α8 helix, anchoring the rest of the protein, which retains its solution fold [16]. Note that in this study, the unfolded loop, connecting helices α1 and α2, was deleted from the protein construct. This anchored conformation is believed to be involved in the canonical mode of apoptotic inhibition in which Bcl-xL binds BAX through the “BH3 binding groove” [7,17,18,19]. In contrast, no high-resolution structure is available for the membrane-inserted conformation of Bcl-xL. It is known, however, that the protein is refolded on the membrane interface with helix α6 penetrating deep into the bilayer, while helix α1 is released from the fold [13,20]. This α1 helix, also known as the BH4 domain, has been proposed to play the central part in the non-canonical mode of apoptotic inhibition by engaging the target BAX via an alternative protein-protein interface (Figure 1b, left) [21]. We hypothesized that changes in MOM lipid composition modulate the conformational switching between the anchored and inserted conformations, allowing for the use of different modes of BAX inhibition (Figure 1b).

Deciphering the molecular mechanisms of Bcl-xL conformational switching in membrane environments is critical for understanding its role as an apoptotic gatekeeping protein. Several studies suggest that the formation of the inserted conformation of Bcl-xL is associated with the protonation of acidic residues. The role of protonation is also supported by the hydropathy analysis presented in Figure 1c for the protonated (blue) and unprotonated (orange) forms of Bcl-xL. While many variables can affect the protonation in the complex milieu inside the cell, the model in vitro studies traditionally rely on changes in pH to trigger the insertion. Previously, we have demonstrated that the refolding and release of the Bcl-xL N-terminal BH4 helix is linked to the membrane insertion of Bcl-xL through processes that are modulated by membrane lipid composition (Figure 1d). In particular, they are promoted by the presence of the mitochondrial specific lipid cardiolipin [13,20]. In Figure 1d, this is observed as a shift of the titration curves towards a more neutral pH range as a function of cardiolipin concentration. These data do not imply that in vivo, the pH must be very acidic for this transition to occur (in a sense, this is similar to studies of thermal stability of proteins, which utilize high temperatures to assess the propensity to refold). Under in vitro conditions, however, these effects are mimicked by modulating the pH of the sample, as one would use temperature to test folding stability.

**Figure 2:**
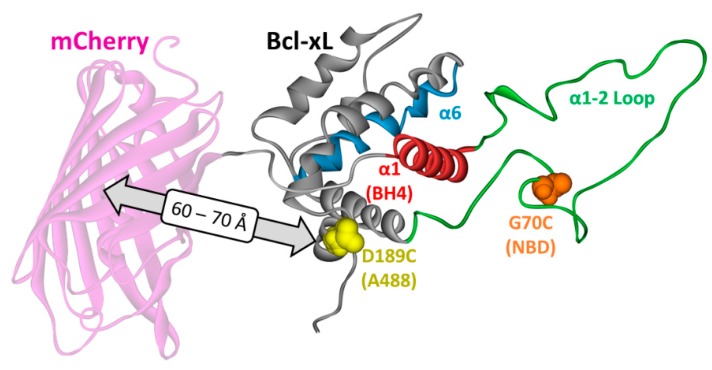
Bcl-xL constructs used in this study. The structure of Bcl-xL solved by NMR [14] is presented as backbone conformation in grey with the following color highlights: hydrophobic helix α6 in blue, BH4 helix α1 (BH4 domain) in red, the loop between α1 and α2 helices in green. The NBD-labeling site in single-Cys G70C mutant is shown in orange. The FRET donor Alexa-Fluor-488-labeling site in the D189C mutant is shown in yellow. The latter construct also had an N-terminally conjugated mCherry fluorescence protein (magenta), to be used as an acceptor in FRET measurements (see text for details).

In order to study conformational switching using fluorescence techniques, we designed the two Bcl-xL mutants illustrated in Figure 2. The release of the BH4 domain was followed by FRET between an Alexa Fluor 488 (A488) donor dye, attached at a single Cys in the D189C mutant, and N-terminus-fused acceptor mCherry fluorescent protein (Figure 2, magenta). The same construct was used in our previously published conformational ensemble study [13], which was complemented here with lifetime measurements and single-molecule fluorescence correlation spectroscopy (FCS) measurements. Membrane interactions of the loop between helices α1 and α2 were studied using the environmentally sensitive probe NBD located at the G70C position. These measurements complement the studies of NBD membrane penetration attached along helix α6 [13]. In both constructs, the C-terminal α8 helix was removed for the following reasons. First, the proper anchoring of Bcl-xL is accomplished by complex targeting machinery in vivo and does not occur with high fidelity in model lipid bilayer vesicles in vitro. Second, the presence of the hydrophobic N-terminal tail substantially complicates the reliability of in vitro spectroscopic studies by promoting protein aggregation [20]. Therefore, in this study, we used a headgroup Bcl-xL construct with a 198–233 a.a. deletion, lacking the C-terminal α8 helix, which is referred from this point on in the text as Bcl-xL (in contrast, the non-truncated protein, which is referred to as full-length Bcl-xL). Note that membrane insertion of the Bcl-xl does not require the presence of the α8 helix [13].

## 2. Materials and Methods

Materials: The fluorescent dyes IANBD-amide (N,N′-Dimethyl-N-(Iodoacetyl)-N′-(7-Nitrobenz-2-Oxa-1,3-Diazol-4-yl)Ethylenediamine) and Alexa Fluor 488-maleimide were obtained from Invitrogen (Carlsbad, CA, USA), while the phospholipids, palmitoyl-oleoyl-phosphatidylcholine (POPC), and 1,1,2,2-tetraoleoyl-cardiolipin (TOCL) were purchased from Avanti Polar Lipids (Alabaster, AL, USA).

Preparation of large unilamellar vesicles (LUV): Chloroform lipid stocks were dried under a nitrogen stream and further dried overnight in high-vacuum. The required volume of 50 mM Na-phosphate buffer, pH 8, was added to the dried lipid films to resuspend them to a final concentration of 20 mM and vortexed. The resuspended samples were extruded using a Mini-Extruder (Avanti Polar Lipids, Alabaster, AL, USA) through 0.1 µm nucleopore polycarbonate membranes (Whatman, Philadelphia, PA, USA) to form the LUV. The vesicle stocks were stored at 4 °C in 50 mM phosphate buffer, pH 8 [22,23].

Cloning, expression, and labeling: The Bcl-xL and mCherry-Bcl-xL mutants were cloned, expressed, and purified, as previously described [13]. The following mutants were employed for our fluorescent studies: 1) Bcl-xL G70C was labeled with NBD for measurements of the α1-2 loop membrane partitioning. 2) Cys-less Bcl-xL for circular dichroism measurements. 3) mCherry-Bcl-xL D189C labeled with Alexa 488 was used for FRET measurements. An additional “donor only” Bcl-xL D189C labeled with Alexa 488 was used for FRET quantification purposes. A molar extinction coefficient of 41,000 M^−1^cm^−1^ at 280 nm was used for the quantification of Bcl-xL protein concentration, while a coefficient of 72,200 M^−1^cm^−1^ at 280 nm and 72,000 M^−1^cm^−1^ at 587 nm was used for mCherry-Bcl-xL. Fluorescent labeling with IANBD and Alexa 488 was performed using a standard labeling protocol for thiol-reactive dyes [24], and the excess dye was removed by gel-filtration in a Superose 6 1 × 30 cm column.

Ensemble fluorescence measurements: Ensemble steady-state fluorescence emission measurements were performed in a Fluorolog FL3-22 steady-state fluorescence spectrometer (Jobin Yvon, Edison, NJ, USA) equipped with double-grating excitation and emission monochromators. The experiments were performed using a 2 × 10 mm cuvette oriented perpendicular to the excitation beam. The sample temperature was maintained constant at 25 °C using a Peltier device from Quantum Northwest (Spokane, WA, USA). All measurements were collected after at least a 15 min equilibration period, after which all spectral measurements were collected with 1 nm steps using a 3 nm slit on the excitation monochromator and 4 nm on the emission monochromator, averaged over 3 scans. NBD fluorescence measurements were collected from 490 to 700 nm using an excitation wavelength of 465 nm. FRET measurements between Alexa Fluor 488 and mCherry dyes were collected between 490–650 nm with a 470 nm excitation wavelength.

**Figure 3:**
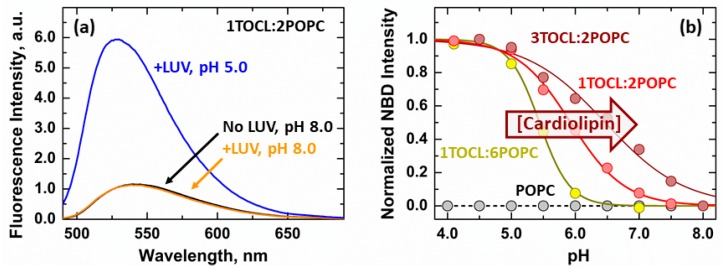
Fluorescence measurements of membrane interaction of the α1-2 loop. (**a**) Acidification of Bcl-xL G70C-NBD in the presence of anionic large unilamellar vesicles (LUV) containing 1TOCL (cardiolipin):2POPC led to a 6-fold increase in fluorescence intensity (blue) compared to measurements at pH 8 (orange), accompanied by a 14 nm blue shift of the NBD emission spectrum from 542 to 528 nm. Both effects are characteristic of the transition of NBD to hydrophobic environments and indicate the protonation-induced membrane association of the α1-2 loop. (**b**) The bilayer interaction of the α1-2 loop was measured as a function of pH in membranes with increasing cardiolipin content. The presence of higher molar ratios of cardiolipin led to more neutral pK_a_ values, indicative of more favorable membrane interactions. The data is presented as the increase in fluorescence intensity associated with the membrane partitioning of G70-NBD measured at 510 nm.

The experiments were performed using 0.3 μM of fluorescently labeled Bcl-xL in 50 mM phosphate buffer at pH 8 and 1 mM LUV. Sample acidification was achieved by the addition of small aliquots of 2.5 M acetate. The quantification of NBD fluorescence intensity changes in Figure 3b was determined at 510 nm. The pH-dependency of the α1-2 loop in the Bcl-xL G70C NBD was calculated by fitting the changes in fluorescence intensity to the following equation [25]:

(1) $$I=\frac{I_{N}+I_{L}\;\left(10^{m\left({\mathrm{pK}}_{a}-\mathrm{pH}\right)}\right)}{1+10^{m\left({\mathrm{pK}}_{a}-\mathrm{pH}\right)}}$$

where I is the fluorescence intensity measured as a function of pH, IN and IL are the limiting intensities at high and low pH, m is the transition slope, and pK_a_ is the negative logarithm of the dissociation constant.

The fluorescence decays of Alexa-Fluor-488/mCherry FRET samples were measured with a FluoTime 200 (PicoQuant, Berlin, Germany) time-resolved fluorescence spectrometer using a standard time-correlated single-photon counting scheme [26]. Samples were excited at 440 nm using a 10 MHz repetition rate subnanosecond pulsed diode laser, LDH 440 (PicoQuant, Berlin, Germany). Fluorescence emission was detected at 520 nm using a PMA-182 photomultiplier (PicoQuant, Berlin, Germany). The emission wavelength was selected using a Sciencetech Model 9030 monochromator. Measurements were performed using 0.3 μM of mCherry-Bcl-xL D189C-Alexa-Fluor-488 or Bcl-xL D189C-Alexa-Fluor-488 in the presence of 1 mM LUV. The fluorescence intensity decay was analyzed using the FluoFit iterative-fitting software (PicoQuant, Berlin, Germany) by subjecting the data to a standard deconvolution procedure. The fitting assumed three exponential components with the shortest lifetime fixed at 0.1 ns. The results were presented as the lifetime, τ_α_, calculated as the amplitude-weighted average lifetime of the two longest components.

FRET analysis: The FRET efficiencies of ensemble steady-state and lifetimes measurements were calculated from changes in fluorescence and lifetime of the donor (Alexa-Fluor 488) in the presence of the acceptor mCherry. The following formulas were employed for the calculations [27]:

(2) $$E=1-\frac{F_{DA}}{F_{D}}$$

(3) $$E=1-\frac{\tau_{DA}}{\tau_{D}}$$

where *F_DA_* and *F_D_* denote to the fluorescence intensity of the donor Alexa-Fluor-488 in the presence or absence of the acceptor mCherry. While *τ_DA_* and *τ_D_* are the corresponding lifetimes of the donor Alexa-Fluor-488 in the presence or absence of a mCherry acceptor. Donor only Bcl-xL D189C samples labeled with Alexa-Fluor-488 dye lacking mCherry were prepared for the donor only measurements.

Single-molecule fluorescence correlation spectroscopy (FCS): FCS FRET measurements were performed, as previously described [28]. Single-molecule fluorescence measurements for FRET experiments were performed with a MicroTime 200 confocal microscope (PicoQuant, Berlin, Germany). The donor Alexa Fluor 488 dye was excited with a pulsed picosecond diode laser LDH-P-C-470 operated at 40 MHz. The resulting fluorescence was split through a 50/50 beam splitter cube onto two Single Photon Avalanche Diodes, SPADs (SPCM—AQR—14, Perkin Elmer Inc., Vaudreuil, Québec, Canada). The fluorescence signal was further split through a set of two filters to separate the signals from the donor (Alexa-Fluor-488) and acceptor (mCherry). An emission band filter (AHF/Chroma: HQ 520/40) was used to detect the Alexa-Fluor-488 donor signal, and a 550 nm long-pass band filter (AHF/Chroma: HQ 550LP) was used for the acceptor mCherry acceptor signal. The high numerical aperture apochromatic water immersion objective (60×, NA 1.2, Olympus), together with the 50 μm confocal pinhole, resulted in a confocal detection volume of 1 fL. The fluorescence signal was detected by applying time-correlated single-photon counting (TCSPC) with a TimeHarp 200 board, and the data was stored in the time-tagged time-resolved mode (TTTR). This allowed the recording of every detected photon with its individual timing and detection channel information. The samples contained 0.1 µM Alexa-Fluor-488 labeled Bcl-xL D189C and 1 mM LUV in 10 mM HEPES buffer + 20 mM NaCl, pH 8. Acidification was achieved by the addition of the appropriate volumes of 0.5 M acetate, and measurements collected after 15 min incubation.

The single-molecule FRET efficiency (smFRET) was calculated from the number of photons detected in the donor (I_D_) and acceptor (I_A_) channels. The smFRET efficiency (E) was calculated from the following formula [29]:

(4) $$E=\frac{I_{A}}{I_{A}+\gamma \cdot I_{D}}$$

where *γ* is a correction factor that takes into account the detection efficiency differences between the two photomultipliers used for the in donor and acceptor channels. The following *γ* parameters were calculated from the integral of the emission spectra of each sample: *γ*_pH 8_ = 3.37, *γ*_pH 7_ = 3.58, and *γ*_pH 6_ = 3.89, respectively.

Circular Dichroism: CD measurements were performed using an upgraded Jasco-720 spectropolarimeter (Japan Spectroscopic Company, Tokyo, Japan). On average, 50 scans were recorded using a 1 mm optical path cuvette. The percent helical folding was estimated assuming the ellipticity at 222 nm corresponds only to α-helical content, following the methodology proposed by Chen et al. [30]:

(5) $$\%\;\mathrm{helical}\;\mathrm{content}=\frac{{\left[\theta \right]}_{222}}{{\left[\theta \right]}_{222}^{Max}\left(1-\frac{k}{n}\right)},\;in\;\deg\cdot cm^{2}\cdot dmol^{-1}$$

where [*θ*]_222_ is the observed ellipticity at 222 nm, [*θ*]_222_^Max^ is the theoretical mean residue ellipticity for an infinitely long helical peptide (−39,500 deg cm^2^ dmol^−1^), *n* is the number of residues (217 in Bcl-xL), and *k* is a wavelength-dependent constant (2.57 at 222 nm) [30].

## 3. Results

### 3.1. Membrane Interactions of the Loop between α1 and α2 Helices

In our previous studies, we used the fluorescence of the environmentally sensitive probe NBD selectively attached to single-Cys residues at various positions along the Bcl-xL sequence to study its membrane partitioning and insertion [13,20]. Here, we used the NBD-labeled G70C Bcl-xL mutant to study the partitioning of the loop between helices α1 and α2 to lipid bilayers (Figure 3). In the absence of membranes, the emission spectrum of Bcl-xL G70C-NBD presented a maximum at 542 nm (Figure 3a, black). The addition of large unilamellar vesicles (LUV) composed of the anionic lipid cardiolipin (TOCL) and the zwitterionic lipid phosphatidylcholine (POPC) at a 1:2 molar ratio had no effect at pH 8 (Figure 3b, orange). This lipid composition represents the maximal cardiolipin content in mitochondria [31,32]. The absence of spectroscopic changes was, therefore, attributed to the lack of Bcl-xL membrane interaction at this pH. Acidification of the sample to pH 5, however, led to a 6-fold increase in fluorescence intensity and a 14 nm blue shift of the band maximum to 528 nm (Figure 3a, blue). These spectroscopic effects were characteristic of the transition of NBD to hydrophobic environments and indicated that the protonation-dependent transition of Bcl-xL to lipid bilayers led to the membrane partitioning of the α1-2 loop.

The membrane insertion of the hydrophobic α6 helix, a characteristic feature of the inserted form of Bcl-xL, is promoted by the mitochondrial specific lipid cardiolipin [13]. For this reason, we tested the effect of cardiolipin on the membrane partitioning of the α1-2 loop by performing pH-titrations in LUV with increasing cardiolipin content. No changes in NBD intensity were observed in zwitterionic bilayers composed solely of POPC lipids (Figure 3b, black). This was consistent with the previously observed requirement of anionic lipids on the membrane insertion and refolding of Bcl-xL [13,20]. Measurements in the presence of cardiolipin containing LUV presented sigmoidal transitions with increasingly more favorable membrane interactions observed in the presence of the higher cardiolipin content. This led to a 1 pH unit difference in the calculated pK_a_ between membranes with the lowest (1TOCL:6POPC, pK_a_ = 5.4 ± 0.1) and highest (3TOCL:2POPC, pK_a_ = 6.4 ± 0.1) cardiolipin content.

Our results showed that the α1-2 loop interacted with cardiolipin-containing bilayers under conditions expected for the inserted form of Bcl-xL. Similar to the effects observed for α6 [13], the interaction of the α1-2 loop was modulated by lipid composition and accentuated in membranes with higher anionic content.

### 3.2. Secondary Structure Changes of Membrane-inserted Bcl-xL

**Figure 4:**
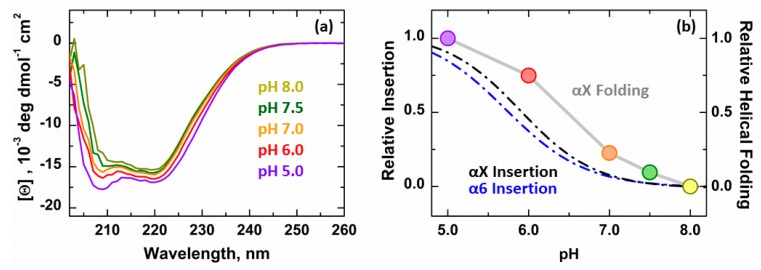
CD measurements of secondary structure changes of Bcl-xL in cardiolipin-containing bilayers. (**a**) The secondary structure of Bcl-xL was measured by circular dichroism in the presence of anionic 1TOCL:2POPC LUV. Under all conditions, the CD spectrum of Bcl-xL presented a double minimum at ~ 209 and 222 nm, characteristic of α-helical conformations. This was consistent with its high X-ray and NMR structures, showing an all α-helical conformation [14]. Inducing the membrane insertion of Bcl-xL through protonation led to a progressive increase in ellipticity at 209 and 222 nm, indicative of a larger α-helical content. (**b**) The relative change in ellipticity at 222 nm, an indicator of α-helical content, at each condition was compared to the protonation-dependent insertion of the α6 helix in blue (Figure 1d, black) and partitioning of the α1-2 loop in black (Figure 3b, red). The helical form of the α1-2 loop in the bilayer is hereby referred to as helix αX. The difference in pH dependence between the insertion and folding data suggested that the bilayer interactions of Bcl-xL did not follow a simple two-state pathway (see also Figure 6).

Circular dichroism (CD) spectroscopy was used to measure the changes in Bcl-xL secondary structure in the presence of membranes under conditions conducive for its insertion. In the presence of 1TOCL:2POPC LUV at pH 8, the CD spectrum of Bcl-xL showed a double minimum at 209 and 222 nm, indicating an α-helical conformation (Figure 4a, yellow), consistent with the X-ray and NMR structures of Bcl-xL in solution [14]. This spectrum was unchanged from the one collected in the absence of membranes. The lack of spectroscopic changes after the addition of LUV was attributed to the previously reported lack of Bcl-xL membrane interactions at this pH [13,20].

The pH-dependent membrane insertion of Bcl-xL (Figure 1d) and the partitioning of its α1-2 loop (Figure 3b) were induced by the progressive acidification of the sample in the presence of LUV. This led to significant increases in ellipticity at 209 and 222 nm (Figure 4a), indicating a larger α-helical content. A total helicity of 40% was calculated in the presence of LUV at pH 8 (Equation (5)) using the determined ellipticity at 222 nm (an α-helical content indicator) of 15,225 × 10^−3^ deg dmol^−1^ cm^2^. This was consistent with the overall helical value of 42% for the NMR structure of Bcl-xL in solution (PDB ID: 1LXL) [14]. A maximal gain in helical content of 4%, corresponding to ~ 10 a.a., was measured at pH 5 (using a total ellipticity of 16,260 × 10^−3^ deg dmol^−1^ cm^2^ at 222 nm). The relative changes in ellipticity at 222 nm are presented in Figure 4b, color-coded to their respective CD spectra in Figure 4a. This data was compared to the relative membrane insertion of Bcl-xL (Figure 4b, black) from Figure 1d, and black and partitioning of the α1-2 loop (Figure 4b, blue) from Figure 3a, red. The discrepancy between these data sets indicated different propensities for each of the respective transitions and a complex multi-step refolding process. Furthermore, it suggested the possibility of multiple stable intermediates in the bilayer.

These measurements showed that protonation in the presence of membranes induced the helical folding of Bcl-xL unstructured loops. Since protonation also induced the membrane insertion of Bcl-xL (Figure 1d), the gain in the secondary structure was likely caused by the partitioning-coupled folding of the α1-2 loop (hereby called helix αX in its folded state) during Bcl-xL membrane insertion.

### 3.3. Ensemble and Single-Molecule FRET Measurements of the BH4 Domain Release in Membrane-Inserted Bcl-xL

**Figure 5:**
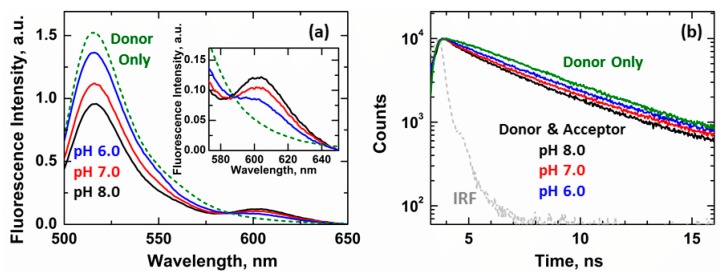
Ensemble FRET measurements of the release of the N-terminal BH4 domain (α1 helix). The release of the BH4 helix was measured by loss of FRET between an N-terminally conjugated mCherry fluorescent protein and the fluorophore Alexa-Fluor-488 (A488) introduced at position D189C in Bcl-xL (Figure 2). (**a**) Steady-state measurements in the presence of 1TOCL:2POPC LUV showed a progressive increase in donor A488 intensity at 518 nm as a function of pH. This was accompanied by a decrease in the acceptor mCherry intensity at 605 nm (insert). These spectral changes were indicative of a loss of FRET between both fluorophores and indicated the increase in distance between donor and acceptor, attributed to the release of the N-terminal BH4 helix. Insert shows a re-scale of the acceptor band. (**b**) Lifetime measurements showed an increase in the fluorescence lifetime of the donor-acceptor samples at increasingly acidic conditions. This was indicative of lower FRET due to a decrease in the interactions between the donor-acceptor pair, consistent with the increase in distance between both fluorophores due to the release of the BH4 helix. The following amplitude average lifetimes were calculated for the donor-acceptor pair in the presence of 1TOCL:2POPC LUV: pH 8 τ_α_ = 2.15 ns (black), pH 7 τ_α_ = 2.76 ns (red), pH 6 τ_α_ = 3.03 ns (blue). The lifetime τ_α_ = 3.42 ns was determined for a donor-only sample in the presence of LUV (green). The internal response function (IRF) of the instrument is indicated in grey.

Recently, we have demonstrated that the membrane insertion of Bcl-xL leads to the release of its N-terminal BH4 helix [13]. The refolding of membranous Bcl-xL was monitored by measuring the loss of FRET in a chimeric protein, where the fluorescent protein mCherry was conjugated at the N-terminus of a Bcl-xL construct labeled with an Alexa-Fluor-488 (A488) dye at position D189C (Figure 2). This event was characterized by a protonation-dependent increase in donor (A488) fluorescence (Figure 5a) at 518 nm and a concomitant decrease in acceptor mCherry intensity at 605 nm (Figure 5a, inset), consistent with previous results [13]. Here, we expanded on this work by performing complimentary lifetime measurements and characterizing this process at the single-molecule level by fluorescence correlation spectroscopy (FCS).

The fluorescence lifetime of a donor-acceptor sample in the presence of 1TOCL:2POPC LUV at pH 8 (non-inserted Bcl-xL) presented an amplitude-averaged lifetime τ_α_ = 2.15 ns (Figure 5b, black). The protonation-induced membrane insertion of Bcl-xL led to the progressively longer lifetimes until they approached the lifetime of a donor-only sample (Figure 5b, green τ_α_ = 3.42 ns). This increase in an averaged lifetime was consistent with our steady-state measurements and caused by the loss of FRET between the donor and acceptor dyes due to the release of the N-terminal BH4 helix.

**Figure 6:**
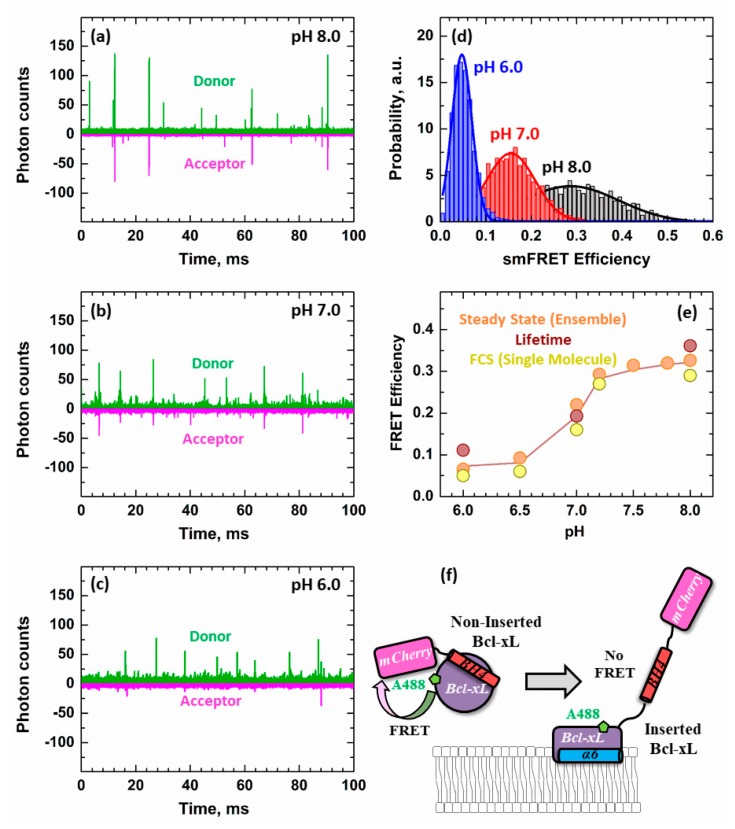
Single-molecule FRET measurements of Bcl-xL refolding. The release of the N-terminal BH4 (α1) helix was inspected at the single-molecule level by fluorescence correlation spectroscopy (FCS) in the presence of 1TOCL:2POPC LUV. Measurements were performed using the same Bcl-xL construct used in Figure 5 between an N-terminally conjugated mCherry fluorescent protein (donor) and acceptor Alexa-Fluor-488 fluorophore introduced at D189C (Figure 2). (**a**–**c**) Representative snapshots of FCS measurements showed individual fluorescence signals detected for each A488 donor (green) or mCherry acceptor (magenta) fluorophore detected. The presence of a spike appearing simultaneously in both acceptor and donor channels indicated positive FRET events between both fluorophores. The number of FRET events decreased proportionally with the pH of the sample. (**d**) The single-molecule FRET efficiency (smFRET) in the sample was calculated using Equation (4) from the number of FRET events detected and fitted to a Gaussian distribution. The loss of FRET was characterized by a progressive shift of the distributions to lower FRET efficiencies as a function of pH. This suggested the presence of multiple stable intermediate conformations during the refolding/membrane insertion of Bcl-xL, each with characteristic FRET distances between the BH4 helix and the rest of Bcl-xL. (**e**) The FRET efficiencies determined by steady-state (Figure 5a), lifetime (Figure 5b), and FCS (Figure 6d) were plotted against experimental pH. (**f**) Schematic of the experimental set-up, indicating the presence of FRET when the acceptor mCherry was close to the donor A488 and the lack of FRET in the refolded/inserted form of Bcl-xL due to the increase in distance between the donor-acceptor pair. The smFRET data indicated that the release of the BH4 helix was not a two-state transition and involved several Bcl-xL intermediates of various compactness.

Single-molecule FCS measurements were performed on these samples to identify the underlying mechanism behind the release of the BH4 helix. Representative traces measured at pH 8, 7, and 6 are shown in Figure 6a–c. A positive FRET event in these measurements was indicated by the simultaneous detection of photons in both the donor (green) and acceptor (magenta) channels while only exciting the donor A488 band. The number of these simultaneous signal spikes was reduced with increasingly acidic conditions due to the loss of FRET, consistent with our steady-state (Figure 5a) and lifetime (Figure 5b) measurements.

The distribution of FRET efficiencies in each single-molecule experiment was calculated using Equation (4) and analyzed with a Gaussian distribution. The observed release of the BH4 helix was not a two-state transition between a globular and a refolded Bcl-xL conformation. Instead, each condition measured produced a distinctive distribution, characterized by a different average FRET efficiency (Figure 6d). This indicated a gradual refolding process with multiple stable intermediates.

The FRET efficiencies calculated from the FCS single-molecule measurements were compared to those determined from steady-state (Equation (2)) and lifetime (Equation (3)) experiments. All FRET measurements agreed with each other. The starting average FRET efficiency of 0.33 at pH 8 (Figure 6e) was consistent with the expected distance between the donor and acceptor fluorophores in the folded construct (Figure 2). All calculated FRET efficiencies decreased as a function of pH and saturated at 0.05 FRET regardless of the technique employed, indicating the unfolding of Bcl-xL and release of BH4.

## 4. Discussion

Apoptosis is crucial for the proper development and function of cell populations in tissues, and its dysregulation impacts many diseases [33,34,35]. Hyperactive apoptosis contributes to neurodegeneration and immunodeficiency, while insufficient apoptosis leads to autoimmunity and cancer, and the ability of cancer cells to avoid apoptosis significantly complicates treatment [36]. The critical step in triggering apoptosis is the permeabilization of the mitochondrial outer membrane (MOMP), which releases apoptotic factors into the cytosol that lead to cell death [37,38]. MOMP is controlled and executed by the numerous proteins of the Bcl-2 family, which include three types: pro-apoptotic pore formers (e.g., BAX, Bak), anti-apoptotic pore inhibitors (e.g., Bcl-xL, BCL-2), and BH3-only regulators (e.g., Bid) [1,2]. These proteins directly interact within the mitochondrial outer membrane (MOM) either to promote or prevent protein conformational changes that lead to the formation of an oligomeric pore [2,9,10,39,40]. Alterations to the lipid composition are also involved in the regulation of apoptosis [41,42,43,44,45].

In spite of the recent advances in solving the structures of the soluble conformations, the exact mechanism of Bcl-2 proteins remains unresolved, primarily because the functionally-important conformations are induced by interactions with the membrane [2,6,40]. A major knowledge gap is the lack of accurate molecular pictures of “protein-protein and protein-lipid interactions that mediate MOMP” [5,9,10]. In this publication, we continued the line of studies [13,20], aiming at bridging this knowledge gap for Bcl-xL.

**Figure 7:**
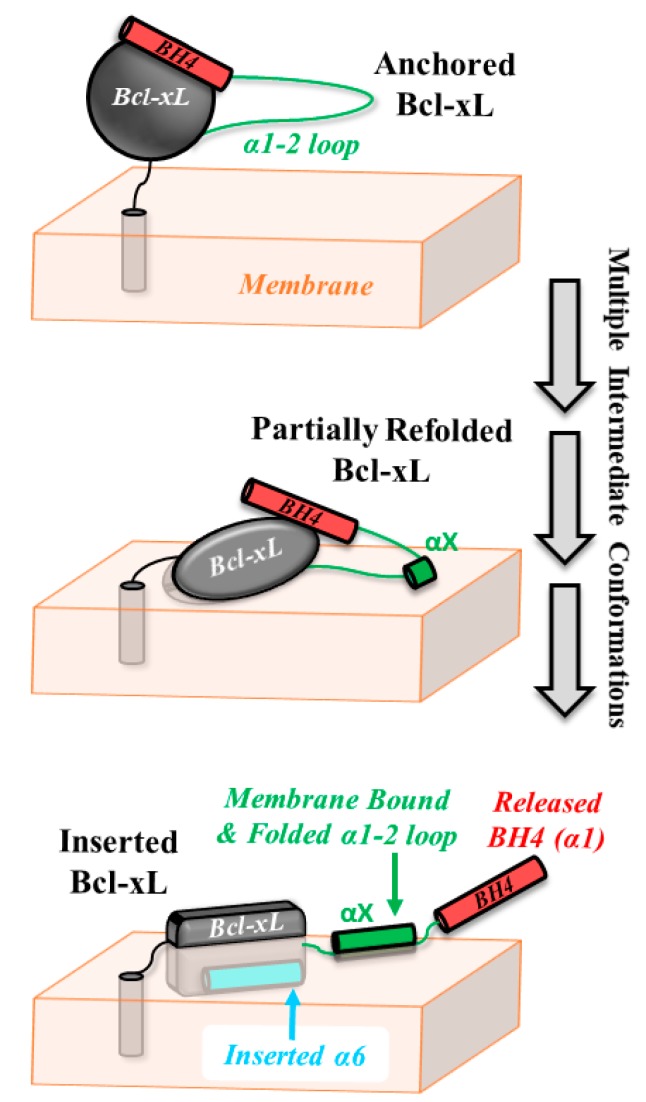
Schematic representation of the conformational switching pathway between membrane-anchored and inserted Bcl-xL. After the initial targeting to the MOM via a yet to be fully understood mechanism, Bcl-xL resides in the conformation closely resembling its solution fold, anchored by a single transmembrane helix α8 [16] (top panel). This anchored conformation is distinctly different from the membrane-inserted one (lower panel), characterized by the refolded secondary and tertiary structure, bilayer penetration of various segments (e.g., α6 helix resides about 15 Å from bilayer center [13], and the release of the regulatory BH4 domain (α1 helix) [13]. In this study, we demonstrated that the originally disordered loop between helices α1 and α2 gained helical structure (helix αX) and interacted with the membrane (Figure 3 and Figure 4). Our single-molecule FRET measurements indicated that the insertion transition contained several intermediate states of different compactness (Figure 6). Lipid composition (notably the presence of cardiolipin and other anionic lipids) modulates the propensity of Bcl-xL to undergo protonation-dependent insertion. We hypothesized that conformational switching between the anchored and the inserted conformations of Bcl-xL results in functional switching between canonical and non-canonical (BH4-dependent) modes of apoptotic inhibition (Figure 1b).

The apoptotic inhibitor Bcl-xL prevents BAX from forming high-order oligomers on the membrane [8], presumably by making a heterodimeric complex. Two non-exclusive models of inhibition have been developed from co-crystallization of regulatory domains with soluble conformations of partner proteins: (1) a canonical mode in which the anti-apoptotic protein captures the BH3 domain of BAX to prevent its homodimerization [19] and (2) a novel, non-canonical mode, in which the BH4 domain of anti-apoptotic protein engages BAX to prevent its activation [21]. The only structural features known of the membrane inserted form of Bcl-xL are the deep interfacial topology of the central hydrophobic α6 helix and the release of the N-terminal BH4 helix [13]. Our results, summarized in Figure 7, show that these processes were accompanied by a gain in helical content (Figure 4), presumably due to the folding of the loop between helixes α1 and α2 (Figure 3).

From our CD measurements (Figure 4), we estimated that upon membrane insertion, the helical content of Bcl-xL increased by the equivalent of a 10-residue segment, which we referred to as helix αX. The only place in the sequence where this could occur was in the extended loop, which we also demonstrated to interact with the membrane (Figure 3). This was consistent with the general thermodynamic principles of partitioning-folding coupling that govern interfacial membrane interactions of proteins and peptides [46]. Notably, upon acidification, the gain in helicity appeared to be happening earlier (i.e., at more neutral pH) than the insertion (Figure 4b). This difference between pH dependencies of the folding and insertion events provided the initial evidence that the insertion process was not a simple two-state transition.

Our single-molecule FRET data on the release of the BH4 domain clearly demonstrated the existence of intermediates of various compactness (Figure 6). The FRET efficiency histogram at pH 8 was centered at 0.3, which was consistent with the structural model presented in Figure 2. In the case of a two-state transition between a folded and completely unfolded state, the histogram for the partial transition would contain a strong component of the folded state, which was not observed at pH 7 or 6. Instead, the entire profile gradually shifted towards low efficiencies, indicating the presence of intermediate states along the insertion/unfolding pathway of Bcl-xL. Note that acidification in the absence of membranes did not lead to changes in FRET and that lipid composition was a factor in this membrane-controlled refolding.

We summarized the results reported here and those from the literature in Figure 7, depicting the conformational changes along the pathway between the anchored (top panel) and inserted conformations of Bcl-xL (bottom panel). We proposed that lipids played a central role in the conformational switching of Bcl-xL, which, in turn, was related to changes in the mode of apoptotic inhibition (Figure 1b). The physiological advantages of the switching between canonic and non-canonic BH4-dependent inhibition of MOMP were not immediately obvious. One could speculate, however, that the reason might be related to the need to counter the cardiolipin-dependent recruitment of BAX to MOM. The release of the BH4 helix would increase the radius of action of Bcl-xL inhibition, while membrane targeting of the loop might direct this action toward membrane-bound BAX. Thus, further studies are needed to establish the structural, thermodynamic, and functional aspects of membrane-modulated conformational switching in Bcl-xL and its role in apoptotic regulation.
